# Risk of fracture in men with prostate cancer on androgen deprivation therapy: a population-based cohort study in New Zealand

**DOI:** 10.1186/s12885-015-1843-3

**Published:** 2015-11-02

**Authors:** Alice Wang, Zuzana Obertová, Charis Brown, Nishi Karunasinghe, Karen Bishop, Lynnette Ferguson, Ross Lawrenson

**Affiliations:** 1 Waikato Clinical School, University of Auckland, Hamilton, New Zealand; 2 Discipline of Nutrition, University of Auckland, Auckland, New Zealand; 3 Auckland Cancer Society Research Centre, University of Auckland, Auckland, New Zealand

**Keywords:** Androgen deprivation therapy, Prostate cancer, Anti-androgens, Orchiectomy, Fracture

## Abstract

**Background:**

Androgen deprivation therapy (ADT) administered as a prostate cancer treatment is known to exert multiple side effects including bone deterioration leading to bone fracture. The current analysis is to evaluate the burden of fracture risk in the New Zealand prostate cancer (PCa) population treated with ADT, and to understand the subsequent risk of mortality after a fracture.

**Methods:**

Using datasets created through linking records from the New Zealand Cancer Registry, National Minimal Dataset, Pharmaceutical Collection and Mortality Collection, we studied 25,544 men (aged ≥40 years) diagnosed with PCa between 2004 and 2012. ADT was categorised into the following groups: gonadotropin-releasing hormone (GnRH) agonists, anti-androgens, combined androgen blockade (GnRH agonists plus anti-androgens), bilateral orchiectomy, and bilateral orchiectomy plus pharmacologic ADT (anti-androgens and/or GnRH agonists).

**Results:**

Among patients receiving ADT, 10.8 % had a fracture compared to 3.2 % of those not receiving ADT (p < 0.0001). After controlling for age and ethnicity, the use of ADT was associated with a significantly increased risk of any fracture (OR = 2.83; 95 % CI 2.52–3.17) and of hip fracture requiring hospitalisation (OR = 1.82; 95 % CI 1.44–2.30). Those who received combined androgen blockade (OR = 3.48; 95 % CI 3.07–3.96) and bilateral orchiectomy with pharmacologic ADT (OR = 4.32; 95 % CI 3.34–5.58) had the greatest risk of fracture. The fracture risk following different types of ADT was confounded by pathologic fractures and spinal cord compression (SCC). ADT recipients with fractures had a 1.83-fold (95 % CI 1.68–1.99) higher mortality risk than those without a fracture. However, after the exclusion of pathologic fractures and SCC, there was no increased risk of mortality.

**Conclusions:**

ADT was significantly associated with an increased risk of any fracture and hip fracture requiring hospitalisation. The excess risk was partly driven by pathologic fractures and SCC which are associated with decreased survival in ADT users. Identification of those at higher risk of fracture and close monitoring of bone health while on ADT is an important factor to consider. This may require monitoring of bone density and bone marker profiles**.**

## Background

Prostate cancer (PCa) is the most commonly registered male cancer and the third leading cause of cancer deaths for New Zealand men, making up 27.3 % of all male cancer registrations and 12.6 % of male cancer deaths in 2011 [1]. Androgen deprivation therapy (ADT) through orchiectomy (surgical castration) or treatment with gonadotropin-releasing hormone (GnRH) agonists (medical castration), or anti-androgens is the mainstay of treatment for metastatic prostate cancer. However, ADT is becoming more commonly used in earlier stages of the disease, particularly as an adjunct to radiation therapy in high-risk localised or locally advanced disease. Survival advantage has been shown in both of these situations [2, 3]. Studies also indicate that ADT together with radiation therapy have a better survival advantage compared to ADT alone especially in patients with locally advanced disease [4]. ADT is also used for the treatment of biochemical relapse (rise in prostate-specific antigen level) after the failure of primary treatment, and as primary therapy for men with localized disease who are unable or unwilling to undergo radical prostatectomy or radiation therapy [2, 5].

Use of ADT, both GnRH agonists and orchiectomy, results in hypogonadism, which is associated with multiple adverse effects including loss of libido, hot flushes, erectile dysfunction, insulin resistance, dyslipidemia, anaemia, fatigue and accelerated bone loss [2, 6–9]. ADT has been associated with an increased risk of bone fracture, as reported in retrospective cohort studies [10–12]. However, few studies have examined the effect of different types of ADT or whether anti-androgens have a different effect on fracture rates. In addition, fractures are known to be associated with increased mortality risk [13, 14], and thus may be an important marker of prognosis in older men with PCa.

We aimed to examine the fracture burden of ADT in New Zealand PCa population. We also evaluated the association between different types of ADT and risk of fractures requiring hospitalisation in the New Zealand PCa population, and the subsequent mortality risk following a fracture.

## Methods

We identified 26,237 men diagnosed with PCa between 2004 and 2012 from the New Zealand Cancer registry (NZCR, http://www.health.govt.nz/nz-health-statistics/national-collections-and-surveys/collections/new-zealand-cancer-registry-nzcr). NZCR has a collection of data since 1948 on all new cases of malignant cancers excluding squamous cell carcinoma and basal cell carcinoma of the skin. The NZCR identifies individuals by their National Health Index (NHI) number, a unique identifier assigned to every person at first contact with the NZ health system. Patients with PCa morphology not consistent with adenocarcinoma (n = 89), and those diagnosed before the age of 40 were excluded (n = 7). We further excluded those who were diagnosed at death (n = 597), leaving a total study population of 25,544 men for analysis. The NZCR data included year of diagnosis, date of birth, age at diagnosis, extent of disease at diagnosis and ethnicity. We divided the study population into age cohorts: 40–49, 50–59, 60–69, 70–79 and ≥ 80 years at PCa diagnosis. The extent of disease at diagnosis is coded as B (localised), C (invasion of adjacent tissue or organs), D (invasion of regional lymph nodes), E (distant metastasis), and F (unknown) in the NZCR. We grouped extent C and D under the category ‘locally advanced’. However, staging data was limited and more than 70 % of the men selected for our study were recorded as ‘Unknown’.

For the purpose of our study, ADT treatment among patients was categorised into one of the five groups: 1) GnRH agonists, 2) anti-androgens, 3) combined androgen blockade (CAB) consisting of GnRH agonists and anti-androgens, 4) bilateral orchiectomy, and 5) bilateral orchiectomy plus pharmacologic ADT (anti-androgens and/or GnRH agonists). By using a unique encrypted number derived from the NHI number, we linked the men identified from the NZCR to the Pharmaceutical Collection (Pharms), which contains records of all subsidised medications dispensed in New Zealand community pharmacies. The Pharms includes the date of dispensing, medication name, quantity and dosage. We extracted data on the usage of GnRH agonists (goserelin acetate, leuprorelin) and anti-androgens (bicalutamide, flutamide, cyproterone acetate). As some of the men had their cancer registered years after the actual diagnosis, the actual date of diagnosis for patients who received pharmacologic ADT was recorded as the date when the first ADT was dispensed or the diagnosis date recorded on NZCR, whichever was earlier. In addition, we also extracted data on use of bisphosphonates (alendronate, zoledronic acid, etidronate, pamidronate). Any record of bilateral orchiectomy was identified through International Classification of Diseases (ICD) 10 codes and extracted from the National Minimum Dataset (NMDS), which contains records of hospital admissions in all public and many private hospitals in New Zealand. In addition, men identified from NZCR were also linked to the Mortality Collection, which registers all deaths occurring in New Zealand.

The study outcome was fracture requiring hospitalisation, identified through linkage to the NMDS. We used the following ICD-10 codes to identify fractures: Accidental fracture (codes S12, S22, S32, S42, S52, S62, S72, S82, S92), pathologic fracture (codes M804, M808, M809, M844, M907, M485, M495), and spinal cord compression (SCC, code G952). Patients were followed for fracture and overall survival until 31 December 2012 and 24 April 2013, respectively.

We used binary logistic regression analysis to calculate adjusted odds ratios (ORs), with 95 % confidence intervals (CIs), for the effect of ADT on fracture risk. The Kaplan-Meier method was used to estimate the fracture-free survival and the 6-month and 12-month survival of ADT users by fracture status. Cox proportional hazard regression model adjusted for age at diagnosis (continuous variable) and ethnicity was used to calculate mortality hazard ratio (HR) and to generate a survival curve by fracture occurrence among ADT users. All analyses were carried out using SAS (V9.2 SAS Institute, Cary, NC, USA).

Ethical approval for this study was obtained from the New Zealand Northern ‘B’ Ethics Committee (Ref. No. MEC/11/EXP/044/AM01). As this study used encrypted data, no informed consent was needed.

## Results

A total of 25,544 men diagnosed with PCa between 2004 and 2012 in New Zealand were included in the analysis. Table 1 summarises the ethnicity, age at diagnosis, year of PCa diagnosis, extent of spread and bisphosphonate intake by use of different types of ADT. Most men were diagnosed between the ages of 60 and 79 years (68.6 %). The mean age of the cohort at PCa diagnosis was 68 (SD = 9.4) years of age.

**Table 1:** Demographic characteristics of men diagnosed with prostate cancer by use of different types of ADT

	All patients		GnRH agonists plus antiandrogens		GnRH agonist only		Anti-androgen monotherapy		Orchiectomy only		Orchiectomy plus pharmacologic ADT		No ADT (%)	
	*N*	%	*n*	%	*n*	%	*n*	%	*n*	%	*n*	%	*n*	%
Age at diagnosis														
40–49	470	1.8	43	9.1	11	2.3	8	1.7	0	0.0	0	0.0	408	86.8
50–59	4388	17.2	454	10.3	216	4.9	120	2.7	9	0.2	30	0.7	3559	81.1
60–69	10,169	39.8	1440	14.2	905	8.9	397	3.9	33	0.3	107	1.1	7287	71.7
70–79	7363	28.8	1835	24.9	946	12.8	491	6.7	119	1.6	218	3.0	3754	51.0
80+	3154	12.3	1012	32.1	320	10.1	429	13.6	107	3.4	127	4.0	1159	36.7
Ethnicity														
European	20,844	81.6	3870	18.6	1923	9.2	1184	5.7	233	1.1	413	2.0	13,221	63.4
Maori	1345	5.3	353	26.2	136	10.1	73	5.4	23	1.7	40	3.0	720	53.5
Pacific_Island	649	2.5	165	25.4	59	9.1	63	9.7	7	1.1	16	2.5	339	52.2
Asian	468	1.8	88	18.8	58	12.4	35	7.5	3	0.6	4	0.9	280	59.8
Other	95	0.4	17	17.9	10	10.5	7	7.4	1	1.1	0	0.0	60	63.2
Non-specify	2143	8.4	291	13.6	212	9.9	83	3.9	1	0.0	9	0.4	1547	72.2
Year of PCa diagnosis														
2004	2715	10.6	483	17.8	203	7.5	271	10.0	59	2.2	107	3.9	1592	58.6
2005	2503	9.8	466	18.6	196	7.8	239	9.5	51	2.0	73	2.9	1478	59.0
2006	2446	9.6	505	20.6	224	9.2	181	7.4	41	1.7	79	3.2	1416	57.9
2007	2892	11.3	570	19.7	301	10.4	170	5.9	39	1.3	66	2.3	1746	60.4
2008	2896	11.3	606	20.9	335	11.6	146	5.0	23	0.8	55	1.9	1731	59.8
2009	3298	12.9	655	19.9	328	9.9	130	3.9	23	0.7	52	1.6	2110	64.0
2010	2890	11.3	558	19.3	268	9.3	123	4.3	18	0.6	33	1.1	1890	65.4
2011	2918	11.4	514	17.6	307	10.5	87	3.0	8	0.3	11	0.4	1991	68.2
2012	2986	11.7	427	14.3	236	7.9	98	3.3	6	0.2	6	0.2	2213	74.1
Extent of disease at diagnosis														
Localised	3810	14.9	51	1.3	32	0.8	63	1.7	16	0.4	10	0.3	3638	95.5
Regional spread	1770	6.9	314	17.7	163	9.2	69	3.9	8	0.5	26	1.5	1190	67.2
Metastatic	1341	5.2	673	50.2	77	5.7	227	16.9	38	2.8	112	8.4	214	16.0
Unknown	18,623	72.9	3746	20.1	2126	11.4	1086	5.8	206	1.1	334	1.8	11,125	59.7
Bisphosphonates														
Yes	1301	5.1	398	30.6	166	12.8	96	7.4	39	3.0	47	3.6	555	42.7
No	24,243	94.9	4386	18.1	2232	9.2	1349	5.6	229	0.9	435	1.8	15,612	64.4

*ADT* androgen deprivation therapy, *GnRH* gonadotropin-releasing hormone

Overall, 9377 of the 25,544 patients (36.7 %) received ADT at some point during follow-up. Among patients who received ADT, 2398 (25.6 %) received GnRH agonists, 1445 (15.4 %) received anti-androgens, 4784 (51 %) received CAB consisting of GnRH agonists and anti-androgens, 268 (2.9 %) underwent orchiectomy alone, and 482 (5.1 %) underwent orchiectomy plus pharmacologic ADT. The rate of use of anti-androgen monotherapy, CAB consisting of GnRH agonists and anti-androgens, orchiectomy and orchiectomy plus pharmacologic ADT increased with increasing age. Compared to European men, Maori and Pacific men were more likely to receive ADT (36.6 % vs 46.5 %; P < 0.0001, and vs 47.8 %; P < 0.0001, respectively). In addition, the use of bisphosphonates was analysed in this cohort. We identified a total of 1301 (5.1 %) patients who received bisphosphonates. Fifty seven percent of these were given to patients who received ADT. The rate of use of bisphosphonates increased with increasing age.

From the entire cohort, 1538 (6.0 %) patients experienced at least one fracture that required hospitalisation (Table 2). Among these patients, 43.3 % had a pathologic fracture and/or SCC. A total of 1014 patients (10.8 %) who received ADT had experienced a fracture compared with 524 patients (3.2 %) not receiving ADT (p < 0.0001). Hip fracture occurred in 218 (2.3 %) patients who received ADT compared to 120 (0.7 %) not receiving ADT (p < 0.0001). Table 3 shows the fracture frequency among ADT users and nonusers by extent of disease. The rates of fractures among ADT users and nonusers were 5.8 and 1.7 % for patients with localised disease (P = 0.0001), and 19.6 and 5.1 % for patients with locally advanced or metastatic disease (P < 0.0001), respectively. We also observed a significant difference in hip fracture rates in patients with localised disease (1.2 % vs 0.2 %, P = 0.0104) and in patients with locally advanced or metastatic disease (2.5 % vs 0.4 %, P < 0.0001). The rates of vertebral fracture were significantly different in patients with localised disease (1.2 % vs 0.3 %, P = 0.0423), but not in patients with locally advanced or metastatic disease (Table 3).

**Table 2:** Frequency of fractures among ADT users and nonusers

	Any fractures (*N* = 1538)	Hip fractures (*N* = 338)	Vertebral fractures (*N* = 273)
No ADT (*n* = 16,167)	524 (3.2 %)	120 (0.7 %)	136 (0.8 %)
Any ADT (*n* = 9377)	1014 (10.8 %)	218 (2.3 %)	137 (1.5 %)
GnRH agonists + Anti-androgens	611 (60.3 %)	110 (50.5 %)	72 (52.6 %)
GnRH agonist only	130 (12.8 %)	43 (19.7 %)	25 (18.2 %)
Anti-androgen only	147 (14.5 %)	43 (19.7 %)	22 (16.0 %)
Orchiectomy alone	38 (3.7 %)	10 (4.6 %)	9 (6.6 %)
Orchiectomy + pharmacologic ADT	88 (8.7 %)	12 (5.5 %)	9 (6.6 %)

*ADT* androgen deprivation therapy, *GnRH* gonadotropin-releasing hormone

**Table 3:** Frequency of fractures among ADT users and nonusers by extent of disease

	Any fracture	Hip fracture	Vertebral fracture
Localised			
No ADT (*N* = 3638)	63 (1.7 %)	7 (0.2 %)	10 (0.3 %)
Any ADT (*N* = 172)	10 (5.8 %)	2 (1.2 %)	2 (1.2 %)
Locally advanced or Metastatic			
No ADT (*N* = 1404)	71 (5.1 %)	6 (0.4 %)	16 (1.1 %)
Any ADT (*N* = 1707)	335 (19.6 %)	43 (2.5 %)	19 (1.1 %)
Unknown			
No ADT (*N* = 11,125)	390 (3.5 %)	107 (1.0 %)	110 (1.0 %)
Any ADT (*N* = 7498)	669 (8.9 %)	173 (2.3 %)	116 (1.6 %)

*ADT* androgen deprivation therapy

We did not observe a statistically significant difference in fracture risk between European and other ethnic groups. After adjusting for age at diagnosis and excluding pathologic fractures and SCC, Maori and Pacific men had a 36 and 55 % reduced risk of any fracture compared to European men, respectively (OR = 0.64; 95 % CI 0.44–0.92 and OR = 0.45; 95 % CI 0.24–0.81, respectively). In addition, Pacific men had a 75 % lower risk of hip fracture than European men (adjusted OR = 0.25; 95 % CI 0.06–1.02) (Table 4). Compared to patients aged <65 years, older patients had a 1.95-fold and 6.22-fold increase in risk of any fracture (95 % CI 1.73–2.20) and hip fracture (95 % CI 4.22–9.17), respectively (Table 4).

**Table 4:** Risk of fracture by ethnicity and age

	Adjusted OR (95 % CI) any fracture	Adjusted OR (95 % CI) accidental fracture	Adjusted OR (95 % CI) hip fracture
Ethnicity^a^			
European	1.00 (Reference)	1.00 (Reference)	1.00 (Reference)
Maori	1.20 (0.96–1.50)	**0.64 (0.44–0.92)**	0.66 (0.35–1.25)
Pacific_Island	0.91 (0.64–1.29)	**0.45 (0.24–0.81)**	0.25 (0.06–1.02)
Asian	0.97 (0.66–1.44)	0.93 (0.57–1.52)	0.48 (0.15–1.50)
Age^b^			
< 65	1.00 (Reference)	1.00 (Reference)	1.00 (Reference)
≥ 65	**1.95 (1.73–2.20)**	**2.23 (1.90–2.62)**	**6.22 (4.22–9.17)**

*OR* odds ratio, *CI* confidence interval

^a^Adjusted for age of diagnosis

^b^Adjusted for ethnicity

Bold indicate significant *p*-value (<0.5)

After controlling for age at diagnosis and ethnicity, the use of ADT was associated with a significantly increased risk of any fracture (OR = 2.83; 95 % CI 2.52–3.17) and of hip fracture requiring hospitalisation (OR = 1.82; 95 % CI 1.44–2.30). When pathologic fractures and SCC were excluded, the overall risk of fracture remained increased, but to a lesser degree (adjusted OR = 1.47; 95 % CI 1.28–1.68) (Table 5). An additional analysis was performed among those with staging data. The use of ADT was associated with the risk of experiencing any fracture and hip fracture in patients with localised disease and locally advanced or metastatic disease (Table 5). However, we did not observe a significant difference in fracture risk, when pathologic fractures and SCC were excluded. We also performed a separate analysis by age (<65 years vs ≥65 years). The use of ADT was associated with increased risk of any fracture in both groups. A higher OR for any fracture was observed in patients aged <65 years (adjusted OR = 4.67; 95 % CI 3.76–5.81). The overall fracture risk was reduced by excluding the pathologic fractures and SCC for patients aged <65 years and those aged ≥65 years. We observed a significant difference in hip fracture risk for patients who aged ≥65 years, but not in patients aged <65 years (Table 5).

**Table 5:** Risk of fracture associated with ADT by extent of disease and age

	Adjusted OR (95 % CI) any fracture	Adjusted OR (95 % CI) accidental fracture	Adjusted OR (95 % CI) hip fracture
**ADT** ^**a**^			
No	1.0 (Reference)	1.0 (Reference)	1.0 (Reference)
Yes	**2.83 (2.52–3.17)**	**1.47 (1.28–1.68)**	**1.82 (1.44–2.30)**
**Extent of disease** ^a^			
Localised			
No ADT	1.0 (Reference)	1.0 (Reference)	1.0 (Reference)
ADT	**3.42 (1.70–6.88)**	1.85 (0.72–4.74)	**6.56 (1.32–32.64)**
Locally advanced or Metastatic			
No ADT	1.0 (Reference)	1.0 (Reference)	1.0 (Reference)
ADT	**3.73 (2.83–4.91)**	1.38 (0.94–2.05)	**3.43 (1.44–8.19)**
Unknown			
No ADT	1.0 (Reference)	1.0 (Reference)	1.0 (Reference)
ADT	**2.21 (1.93–2.52)**	**1.40 (1.20–1.63)**	**1.60 (1.25–2.06)**
**Age** ^**b**^			
< 65			
No ADT	1.0 (Reference)	1.0 (Reference)	1.0 (Reference)
ADT	**4.67 (3.76–5.81)**	**1.50 (1.10–2.05)**	2.02 (0.93–4.39)
≥ 65			
No ADT	1.0 (Reference)	1.0 (Reference)	1.0 (Reference)
ADT	**2.89 (2.55–3.29)**	**1.87 (1.61–2.17)**	**2.53 (1.99–3.22)**

*ADT* androgen deprivation therapy, *OR* odds ratio, *CI* confidence interval

^a^Adjusted for age of diagnosis and ethnicity

^b^Adjusted for ethnicity

Bold indicate significant *p*-value (<0.5)

With respect to different types of ADT, the highest OR for fracture risk was observed in patients who underwent orchiectomy combined with pharmacologic ADT (adjusted OR = 4.32; 95 % CI 3.34–5.58), followed by patients receiving CAB consisting of GnRH agonists and anti-androgens (adjusted OR = 3.48; 95 % CI 3.07–3.96). A higher OR was observed in patients who had orchiectomy alone (adjusted OR = 2.54; 95 % CI 1.76–3.66) than in patients who had anti-androgen alone (adjusted OR = 2.11; 95 % CI 1.72–2.59). The lowest risk was observed in patients treated with GnRH agonists alone (adjusted OR = 1.33; 95 % CI 1.09–1.63) (Table 6). After excluding pathologic fractures and SCC, we did not observe an increased fracture risk in patients who underwent orchiectomy plus pharmacologic ADT and in patients who had GnRH agonists alone. After the exclusion of pathologic fractures and SCC, the adjusted OR was reduced to 2.18 (95 % CI 1.43–3.33) for patients who had orchiectomy alone, 1.60 (95 % CI 1.36–1.88) for patients receiving CAB consisting of GnRH agonists and anti-androgens and 1.37 (95 % CI 1.07–1.77) for those who had anti-androgen alone (Table 6).

**Table 6:** Risk of fracture associated with different types of ADT

	Adjusted OR (95 % CI) any fracture^a^	Adjusted OR (95 % CI) accidental fracture^a^	Adjusted OR (95 % CI) hip fracture^a^
No ADT	1.0 (Reference)	1.0 (Reference)	1.0 (Reference)
GnRH agonists + anti-androgens	**3.48 (3.07–3.96)**	**1.60 (1.36–1.88)**	**1.82 (1.39–2.38)**
GnRH agonist only	**1.33 (1.09–1.63)**	1.17 (0.93–1.47)	**1.70 (1.19–2.42)**
Anti-androgen only	**2.11 (1.72–2.59)**	**1.37 (1.07–1.77)**	**1.81 (1.24–2.63)**
Orchiectomy alone	**2.54 (1.76–3.66)**	**2.18 (1.43–3.33)**	1.88 (0.96–3.67)
Orchiectomy + pharmacologic ADT	**4.32 (3.34–5.58)**	1.39 (0.93–2.07)	1.59 (0.86–2.93)

*ADT* androgen deprivation therapy, *GnRH* gonadotropin-releasing hormone, *OR* odds ratio, *CI* confidence interval

^a^Adjusted for age at diagnosis and ethnicity

Bold indicate significant *p*-value (<0.5)

The use of bisphosphonates was associated with a significantly increased risk of any fracture (adjusted OR = 4.12; 95 % CI 3.56–4.77) and of hip fracture (adjusted OR = 5.20; 95 % CI 4.06–6.67) requiring hospitalisation. Among those with staging data, the use of bisphosphonates was associated with 5.89-fold and 2.03-fold increased risk of any fracture in patients with localised disease (95 % CI 2.81–12.38) and locally advanced or metastatic disease (95 % CI 1.42–2.90), respectively. We did not observe a significant difference in hip fracture risk with the use of bisphosphonates in patients at any stage (Table 7).

**Table 7:** Risk of fracture associated with the use of bisphosphonates

	Adjusted OR (95 % CI) any fracture	Adjusted OR (95 % CI) accidental fracture	Adjusted OR (95 % CI) hip fracture
**Use of bisphosphonate** ^**a**^			
No	1.00 (Reference)	1.00 (Reference)	1.00 (Reference)
Yes	**4.12 (3.56–4.77)**	**4.21 (3.55–5.0)**	**5.20 (4.06–6.67)**
**Extent of disease** ^**a**^			
Localised			
No bisphosphonate	1.00 (Reference)	1.00 (Reference)	1.00 (Reference)
Bisphosphonate	**5.89 (2.81–12.38)**	**5.46 (2.38–12.50)**	5.16 (0.61–43.55)
Locally advanced or Metastatic			
No bisphosphonate	1.00 (Reference)	1.00 (Reference)	1.00 (Reference)
Bisphosphonate	**2.03 (1.42–2.90)**	**1.97 (1.17–3.33)**	1.76 (0.79–3.91)
Unknown			
No bisphosphonate	1.00 (Reference)	1.00 (Reference)	1.00 (Reference)
Bisphosphonate	**4.85 (4.11–5.73)**	**4.62 (3.83–5.56)**	**6.01 (4.61–7.83)**

*OR* odds ratio, *CI* confidence interval

^a^Adjusted for age at diagnosis and ethnicity

Bold indicate significant *p*-value (<0.5)

**Fig. 1:**
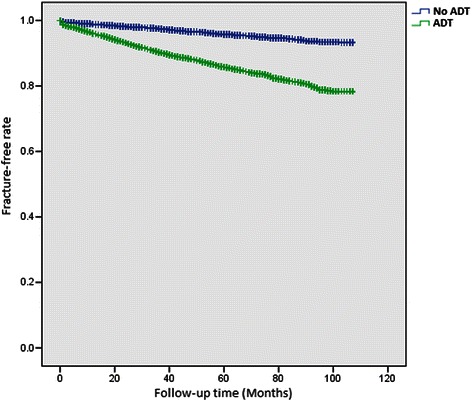
Kaplan-Meier plots of fracture-free survival in ADT users versus nonusers

Figure 1 presents the estimates of fracture-free survival by use of ADT. Men who received ADT had a lower rate of fracture-free survival than men who did not (log-rank test: *P* <0.0001). The 5-year fracture-free survival rate was 85.8 % for ADT users and 95.8 % for nonusers.

**Fig. 2:**
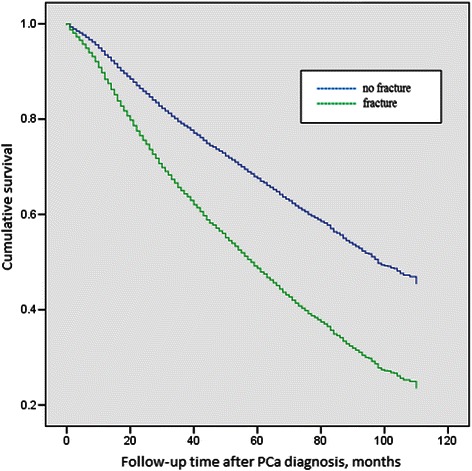
Survival probability among ADT users by status of fracture

Fractures requiring hospitalisation were associated with an increase in overall mortality. The mortality of ADT recipients experiencing any fracture was 4.5 % within 6 months and 13.7 % within 12 months. Figure 2 shows the survival curve derived from Cox proportional hazard regression model among ADT users by fracture status. Among men who received ADT, fracture requiring hospitalisation was associated with a 1.83-fold increase in the rate of mortality (95 % CI, 1.68–1.99), after adjusting for age at diagnosis and ethnicity. However, when pathologic fractures and SCC were excluded, there was no significant association between fractures and mortality risk.

## Discussion

In the present population-based cohort study, the use of ADT was associated with a 2.83-fold increase in risk of fracture requiring hospitalisation. Our results are consistent with previous retrospective cohort studies that reported an association between ADT and fracture risk [10–12, 15, 16]. In a large cohort study that examined the fracture rate in more than 50,000 men who survived at least 5 years after PCa diagnosis, 19.4 % of those who received ADT had a fracture, compared with 12.6 % of those not receiving ADT [10]. In another large retrospective cohort study, men treated with ADT for at least 6 months experienced more fragility fractures than matched controls (9.0 % vs 5.9 %; HR = 1.65; 95 % CI 1.53–1.78) [17]. We also included pathologic fractures for our main analysis, based on evidence that their exclusion can underestimate the fracture burden due to osteoporosis [18]. However, when pathologic fractures and SCC were excluded, the overall OR remained elevated but was reduced to 1.47. The use of ADT was associated with a 1.82-fold increase in risk of hip fracture requiring hospitalisation in the present study. Similarly, a case-control study in Denmark reported that any use of ADT was associated with an OR of 1.9 for hip fracture [19].

With regards to ethnicity, we observed that Maori and Pacific men had a lower risk of any fracture compared to men of European origin. This could be partly due to difference in genetic and lifestyle factors including diet, activity level and body mass. It is known that for the same height and weight, men of Maori and Pacific origin have less fat mass and more lean mass than that of the Europeans [20, 21]. It has been shown that for men each additional kilogram of lean mass is associated with an expected bone mineral density (BMD) increase of 2.48–5.90 mg/cm^2^ while each kilogram of fat mass is associated with 1.48–3.29 mg/cm^2^ increase in BMD across total body, lumbar spine, total hip and femoral neck. This study also shows these associations to be more effective in those within the first tertile of body mass index (BMI; ≤26.5 kg/m^2^), while attenuating at higher BMI. They also noted that it is more appropriate to consider low body weight as a risk factor for osteoporosis rather than considering obesity as a protective factor [22].

We observed increased fracture risk with all types of ADT. A prior population-based study did not observe an association between the use of anti-androgens and risk of fracture requiring hospitalisation [23]. On the contrary, we observed a 2.11-fold increase in fracture risk among patients treated with anti-androgens alone. Although anti-androgens have been shown to maintain or even increase BMD in clinical trials [24–26], it was an independent predictor of increased fracture risk in our study. This could be due to anti-androgen being used mainly as a palliative therapy for older men with fragile bones or being used as a palliative therapy for older men with bone metastases. A claims-based analysis of more than 11,000 men with non-metastatic PCa reported a relative risk of fracture of 1.21 among men treated with GnRH agonists compared with those who were not [12]. S**i**milarly, we observed a 1.33-fold increase in fracture risk for men receiving GnRH agonists. However, the association was confounded by pathologic fractures and SCC. CAB was associated with a 3.48-fold increase in fracture risk in our study. The risk was increased further among patients who underwent orchiectomy and also received pharmacologic ADT. However, when pathologic fractures and SCC were excluded, the OR was decreased for CAB, anti-androgen alone, and orchiectomy, and no increased risk was observed for GnRH agonists alone and orchiectomy plus pharmacologic ADT. These results indicate that some of the excess fracture risk associated with the various forms of ADT may be due to the development of bone metastases. However, as the majority of the men in our study were not staged in NZCR, we were not able to ascertain whether the fracture was due to bone metastases or ADT. After the exclusion of pathologic fractures and SCC, the OR was higher for CAB consisting of GnRH agonists and anti-androgens than anti-androgens alone. This finding suggests a possible additive effect exerted by ADT on both reduction of testosterone level and receptor antagonism. An alternative explanation is that this finding is due to prescribing bias, in which patients at high fracture risk may receive the strongest treatment, such as CAB mentioned above. It is also possible that CAB is prescribed in the late stages of the disease at which there could be hidden metastases causing bone fractures. The risk of fracture after exclusion of pathologic fracture and SCC is the highest in patients treated with orchiectomy alone. This may be partly explained by the irreversible androgen suppression associated with orchiectomy. Alternatively this may be due to factors that cannot be explained by the current available datasets. For instance the negative psychological impact associated with orchiectomy may lead to reduced physical activity level affecting bone health.

We have performed an additional analysis of fracture risk among those with staging data. The use of ADT was associated with 3.42 and 3.73-fold increase in risk of fracture requiring hospitalisation in patients with localised and locally advanced or metastatic disease, respectively. However, when pathologic fractures and SCC were excluded, we did not observe an increased risk. This indicates the associations were confounded by pathologic fracture and SCC. We observed a 3.43-fold increase in hip fracture risk in locally advanced or metastatic disease patients. The risk of hip fracture was increased further to 6.56-fold for localised patients. Thus, the use of ADT has a greater impact on hip fracture risk for patients with localised disease.

Studies have demonstrated that bisphosphonates are effective in preventing bone loss associated with ADT [27]. In the present study, we found that those who received bisphosphonate had a 4.12 and 5.20-fold increase in risk of any fracture and hip fracture, respectively. However, this may be due to prescribing bias, in which patients receiving bisphosphonate already had a bone fracture. In the Randomised Androgen Deprivation and Radiation (RADAR) study, Denham et al assessed the effect of zoledronic acid (ZdA) on BMD and bone fracture in men with locally advanced PCa. The authors did not observe that ZdA had an influence on incidence of bone fracture [28]. Later on, the authors reported that adding 18 months of ZdA for patients receiving only 6 months of androgen suppression increased the risk of bone progression [29]. This indicated that giving bisphosphonate in the absence of androgen suppression is detrimental in terms of cancer progression to bone. In our study, the increased risk of bone fracture associated with use of bisphosphonate may be attributed to a high proportion of patients who were given bisphosphonate in the absence of ADT. In a histomorphometric study on autopsy samples, Morissey et al showed that in castration-resistant prostate cancer (CRPC), ADT induces serious bone loss even in patients treated with bisphosphonate. The authors also showed that bisphosphonate treatment increased bone volume but did not decrease the number of osteoclasts at the site of bone metastasis compared to those who did not receive bisphosphonate [30]. They have also reported observing giant osteoclasts at the bone sites without metastatic lesions in the group who received bisphosphonates. The results from present study may be partly due to the limited efficacy of bisphosphonates to improve bone responses in patients who may be having CRPC.

We also found an increased risk of overall mortality following a fracture in the present study, which is consistent with findings reported from previous studies on fracture and mortality [14, 31]. A cohort study of more than 80,000 PCa patients also found that men who sustained a fracture have a two-fold increase in death rate compared to those without a fracture [32]. However, we did not observe a significant difference in the risk of mortality when pathologic fractures and SCC were excluded. This finding indicates that pathologic fractures and SCC significantly contributed to decreased survival. Indeed, the mortality risk increased 3.31-fold following a pathologic fracture and/or SCC.

Our study has several strengths including a large sample size, a population-based cohort of men given ADT and relatively long follow-up time. There are several limitations of our analysis that should be noted. A major limitation was that majority of patients are not staged in NZCR (>70 %), and that some of the patients may have their cancer registered years after the actual diagnosis. To improve the accuracy of diagnosis date, we adjusted the date of diagnosis to the date when the first ADT was dispensed for patients who had their first ADT prescription before the diagnosis date recorded on the NZCR. As ADT can be commenced some time after diagnosis, the adjustment of diagnosis date may introduce bias. However, this is the best estimation for the date of diagnosis. Another limitation was that many fractures are not recorded in the NMDS, thus fracture risk may be underestimated. For example, fractures of the forearm are usually treated on an outpatient basis, in emergency clinics, and therefore will not be recorded in the NMDS. In addition, vertebral compressions are often asymptomatic, and are not diagnosed and recorded in the NMDS. Another limitation is the lack of data on certain risk factors of fracture, such as smoking status, alcohol consumption, use of corticosteroids, BMI, and family history of fracture, thus we were not able to adjust our analysis for these important potential confounders.

## Conclusions

In the present population-based cohort study, we confirmed the association between ADT and fracture risk in the New Zealand PCa population. The excess fracture risk was partly driven by pathologic fractures and SCC. We believe identification of those at higher risk of fracture and close monitoring of bone health while on ADT in this group is an important factor that needs to be taken into account.

## Abbreviations

ADT: androgen deprivation therapy
BMD: bone mineral density
BMI: body mass index
CAB: combined androgen blockade
CI: confidence interval
CRPC: castration-resistant prostate cancer
GnRH: gonadotropin-releasing hormone
HR: hazard ratio
ICD: international classification of diseases
NMDS: national minimum dataset
NHI: national health Index
NZCR: New Zealand cancer registry
OR: odds ratio
PCa: prostate cancer
Pharms: pharmaceutical collection
SCC: spinal cord compression
ZdA: zoledronic acid

## References

[1] Ministry of Health (2014). Cancer: new registrations and deaths 2011.

[2] Sharifi N, Gulley JL, Dahut WL (2005). Androgen deprivation therapy for prostate cancer. JAMA.

[3] Roach M (2014). Current trends for the use of androgen deprivation therapy in conjunction with radiotherapy for patients with unfavorable intermediate-risk, high-risk, localized, and locally advanced prostate cancer. Cancer.

[4] Schmidt-Hansen M, Hoskin P, Kirkbride P, Hasler E, Bromham N (2014). Hormone and radiotherapy versus hormone or radiotherapy alone for non-metastatic prostate cancer: a systematic review with meta-analyses. Clin Oncol (R Coll Radiol).

[5] Studer UE, Whelan P, Albrecht W, Casselman J, de Reijke T, Hauri D, Loidl W, Isorna S, Sundaram SK, Debois M (2006). Immediate or deferred androgen deprivation for patients with prostate cancer not suitable for local treatment with curative intent: European Organisation for Research and Treatment of Cancer (EORTC) Trial 30891. J Clin Oncol.

[6] Sharifi N, Gulley JL, Dahut WL (2010). An update on androgen deprivation therapy for prostate cancer. Endocr Relat Cancer.

[7] Greenspan SL (2005). Bone loss after initiation of androgen deprivation therapy in patients with prostate cancer. J Clin Endocrinol Metab.

[8] Taylor LG, Canfield SE, Du XL (2009). Review of major adverse effects of androgen-deprivation therapy in men with prostate cancer. Cancer.

[9] Alibhai SMH, Gogov S, Allibhai Z (2006). Long-term side effects of androgen deprivation therapy in men with non-metastatic prostate cancer: a systematic literature review. Crit Rev Oncol Hematol.

[10] Shahinian VB, Kuo Y-F, Freeman JL, Goodwin JS (2005). Risk of fracture after androgen deprivation for prostate cancer. N Engl J Med.

[11] Smith MR, Boyce SP, Moyneur E, Duh MS, Raut MK, Brandman J (2006). Risk of clinical fractures after gonadotropin-releasing hormone agonist therapy for prostate cancer. J Urol.

[12] Smith MR, Lee WC, Brandman J, Wang Q, Botteman M, Pashos CL (2005). Gonadotropin-releasing hormone agonists and fracture risk: a claims-based cohort study of men with nonmetastatic prostate cancer. J Clin Oncol.

[13] Center JR, Nguyen TV, Schneider D, Sambrook PN, Eisman JA (1999). Mortality after all major types of osteoporotic fracture in men and women: an observational study. Lancet.

[14] Bliuc D, Nguyen ND, Milch VE, Nguyen TV, Eisman JA, Center JR (2009). Mortality risk associated with low-trauma osteoporotic fracture and subsequent fracture in men and women. JAMA.

[15] Lau Y-K, Lee E, Prior HJ, Lix LM, Metge CJ, Leslie WD (2009). Fracture risk in androgen deprivation therapy: a Canadian population based analysis. Can J Urol.

[16] Lopez AM, Pena MA, Hernandez R, Val F, Martin B, Riancho JA (2005). Fracture risk in patients with prostate cancer on androgen deprivation therapy. Osteoporos Int.

[17] Alibhai SMH, Duong-Hua M, Cheung AM, Sutradhar R, Warde P, Fleshner NE, Paszat L (2010). Fracture types and risk factors in men with prostate cancer on androgen deprivation therapy: a matched cohort study of 19,079 men. J Urol.

[18] Curtis JR, Taylor AJ, Matthews RS, Ray MN, Becker DJ, Gary LC, Kilgore ML, Morrisey MA, Saag KG, Warriner A (2009). “Pathologic” fractures: should these be included in epidemiologic studies of osteoporotic fractures?. Osteoporos Int.

[19] Abrahamsen B, Nielsen MF, Eskildsen P, Andersen JT, Walter S, Brixen K (2007). Fracture risk in Danish men with prostate cancer: a nationwide register study. BJU Int.

[20] Rush EC, Freitas I, Plank LD (2009). Body size, body composition and fat distribution: comparative analysis of European, Maori, Pacific Island and Asian Indian adults. Br J Nutr.

[21] Rush E, Plank L, Chandu V, Laulu M, Simmons D, Swinburn B, Yajnik C (2004). Body size, body composition, and fat distribution: a comparison of young New Zealand men of European, Pacific Island, and Asian Indian ethnicities. N Z Med J.

[22] Zhu K, Hunter M, James A, Lim EM, Walsh JP (2015). Associations between body mass index, lean and fat body mass and bone mineral density in middle-aged Australians: The Busselton Healthy Ageing Study. Bone.

[23] Thorstenson A, Bratt O, Akre O, Hellborg H, Holmberg L, Lambe M, Bill-Axelson A, Stattin P, Adolfsson J (2012). Incidence of fractures causing hospitalisation in prostate cancer patients: results from the population-based PCBaSe Sweden. Eur J Cancer.

[24] Smith MR, Goode M, Zietman AL, McGovern FJ, Lee H, Finkelstein JS (2004). Bicalutamide monotherapy versus leuprolide monotherapy for prostate cancer: effects on bone mineral density and body composition. J Clin Oncol.

[25] Wadhwa VK, Weston R, Mistry R, Parr NJ (2009). Long-term changes in bone mineral density and predicted fracture risk in patients receiving androgen-deprivation therapy for prostate cancer, with stratification of treatment based on presenting values. BJU Int.

[26] Sieber PR, Keiller DL, Kahnoski RJ, Gallo J, McFadden S (2004). Bicalutamide 150 mg maintains bone mineral density during monotherapy for localized or locally advanced prostate cancer. J Urol.

[27] Serpa Neto A, Tobias-Machado M, Esteves MAP, Senra MD, Wroclawski ML, Fonseca FLA, Dos Reis RB, Pompeo ACL, Giglio AD (2012). Bisphosphonate therapy in patients under androgen deprivation therapy for prostate cancer: a systematic review and meta-analysis. Prostate Cancer Prostatic Dis.

[28] Denham JW, Nowitz M, Joseph D, Duchesne G, Spry NA, Lamb DS, Matthews J, Turner S, Atkinson C, Tai K-H (2014). Impact of androgen suppression and zoledronic acid on bone mineral density and fractures in the Trans-Tasman Radiation Oncology Group (TROG) 03.04 Randomised Androgen Deprivation and Radiotherapy (RADAR) randomized controlled trial for locally advanced prostate cancer. BJU Int.

[29] Denham JW, Joseph D, Lamb DS, Spry NA, Duchesne G, Matthews J, Atkinson C, Tai KH, Christie D, Kenny L (2014). Short-term androgen suppression and radiotherapy versus intermediate-term androgen suppression and radiotherapy, with or without zoledronic acid, in men with locally advanced prostate cancer (TROG 03.04 RADAR): an open-label, randomised, phase 3 factorial trial. Lancet Oncol.

[30] Morrissey C, Roudier MP, Dowell A, True LD, Ketchanji M, Welty C, Corey E, Lange PH, Higano CS, Vessella RL (2013). Effects of androgen deprivation therapy and bisphosphonate treatment on bone in patients with metastatic castration-resistant prostate cancer: results from the University of Washington Rapid Autopsy Series. J Bone Miner Res.

[31] Haentjens P, Magaziner J, Colon-Emeric CS, Vanderschueren D, Milisen K, Velkeniers B, Boonen S (2010). Meta-analysis: excess mortality after hip fracture among older women and men. Ann Intern Med.

[32] Beebe-Dimmer JL, Cetin K, Shahinian V, Morgenstern H, Yee C, Schwartz KL, Acquavella J (2012). Timing of androgen deprivation therapy use and fracture risk among elderly men with prostate cancer in the United States. Pharmacoepidemiol Drug Saf.

